# NRF2 and PPAR-γ Pathways in Oligodendrocyte Progenitors: Focus on ROS Protection, Mitochondrial Biogenesis and Promotion of Cell Differentiation

**DOI:** 10.3390/ijms21197216

**Published:** 2020-09-29

**Authors:** Chiara De Nuccio, Antonietta Bernardo, Carmen Troiano, Maria Stefania Brignone, Mario Falchi, Anita Greco, Michela Rosini, Filippo Basagni, Cristina Lanni, Melania Maria Serafini, Luisa Minghetti, Sergio Visentin

**Affiliations:** 1 Research Coordination and Support Service, Istituto Superiore di Sanità, 00161 Rome, Italy; chiara.denuccio@iss.it (C.D.N.); luisa.minghetti@iss.it (L.M.); 2 National Center for Research and Preclinical and Clinical Evaluation of Drugs, Istituto Superiore di Sanità, 00161 Rome, Italy; antonietta.bernardo@iss.it (A.B.); anita.greco@iss.it (A.G.); 3 Department of Cell Biology and Neurosciences, Istituto Superiore di Sanità, 00161 Rome, Italy; carmentroiano@alice.it; 4 Department of Neuroscience, Istituto Superiore di Sanità, 00161 Rome, Italy; mariastefania.brignone@iss.it; 5 National Research Center on HIV/AIDS, Istituto Superiore di Sanità, 00161 Rome, Italy; mario.falchi@iss.it; 6 Department of Pharmacy and Biotechnology, University of Bologna, 40126 Bologna, Italy; michela.rosini@unibo.it (M.R.); filippo.basagni2@unibo.it (F.B.); 7 Department of Drug Sciences, University of Pavia, 27100 Pavia, Italy; cristina.lanni@unipv.it (C.L.); melania.serafini@gmail.com (M.M.S.)

**Keywords:** PPAR-γ, NRF2, oligodendrocytes, mitochondria, DMF, pioglitazone

## Abstract

An adequate protection from oxidative and inflammatory reactions, together with the promotion of oligodendrocyte progenitor (OP) differentiation, is needed to recover from myelin damage in demyelinating diseases. Mitochondria are targets of inflammatory and oxidative insults and are essential in oligodendrocyte differentiation. It is known that nuclear factor-erythroid 2-related factor/antioxidant responsive element (NRF2/ARE) and peroxisome proliferator-activated receptor gamma/PPAR-γ response element (PPAR-γ/PPRE) pathways control inflammation and overcome mitochondrial impairment. In this study, we analyzed the effects of activators of these pathways on mitochondrial features, protection from inflammatory/mitochondrial insults and cell differentiation in OP cultures, to depict the specificities and similarities of their actions. We used dimethyl-fumarate (DMF) and pioglitazone (pio) as agents activating NRF2 and PPAR-γ, respectively, and two synthetic hybrids acting differently on the NRF2/ARE pathway. Only DMF and compound 1 caused early effects on the mitochondria. Both DMF and pio induced mitochondrial biogenesis but different antioxidant repertoires. Moreover, pio induced OP differentiation more efficiently than DMF. Finally, DMF, pio and compound 1 protected from tumor necrosis factor-alpha (TNF-α) insult, with pio showing faster kinetics of action and compound 1 a higher activity than DMF. In conclusion, NRF2 and PPAR-γ by inducing partially overlapping pathways accomplish complementary functions aimed at the preservation of mitochondrial function, the defense against oxidative stress and the promotion of OP differentiation.

## 1. Introduction

In the event of myelin damage or loss, occurring in multiple sclerosis and leukodystrophies, remyelination becomes necessary for the recovery and maintenance of nerve functions. During the remyelination process, a population of undifferentiated oligodendrocyte (OL) progenitors (OPs) is recruited to the site of injury to differentiate toward myelin-producing cells. On the other hand, several factors and events not fully disclosed often hinder the process and impede its completion [1,2,3]. In a therapeutic perspective, strategies aimed at stimulating remyelination by targeting OLs are the object of intense research. The aim of such approach would be to reinforce the OP capability to survive environmental stress and to promote differentiation toward myelin-producing cells.

A rapidly evolving literature indicates that mitochondria are crucial for proper myelination to occur and that differentiating OPs show the highest sensitivity to mitochondrial dysfunction [4,5,6] compared to more mature OL stages.

Mitochondria supply the energy, in the form of adenosine triphosphate (ATP), required for the high metabolic rate of differentiating cells, and large amounts of membrane components of myelin, such as cholesterol. The molecules produced by mitochondria (e.g., reactive oxygen species (ROS), ATP, and others) are also involved in intracellular signal transduction and are potentially crucial for OL differentiation [7,8,9]. In keeping with the role of mitochondria in OL differentiation, nuclear and mitochondrial genes coding for mitochondrial proteins are upregulated during specific phases of OL differentiation [4].

Among the endogenous cell processes capable of promoting mitochondrial functions and the endogenous defense mechanisms there are those triggered by the peroxisome proliferator-activated receptor gamma (PPAR-γ) and by the transcription factor nuclear factor-erythroid 2 (NF-E2)-related factor (NRF2) [10,11].

PPAR-γ is a member of the nuclear receptor superfamily and shares with other two isotypes (α and β/δ) the capability to control cell metabolism, differentiation, and immune responses. PPAR-γ is mostly expressed in adipocytes, where it regulates adipogenesis in immune cells and the brain. It can be activated by endogenous compounds, like long-chain fatty acids, and by synthetic agonists, like nonsteroidal anti-inflammatory molecules and insulin-sensitizing agents (e.g., thiazolidinediones). Once activated, it forms heterodimers with retinoic x receptor (RXR) receptors bound to retinoic acid and moves to the nucleus. In the nucleus, it interacts with coactivators, such as the PPAR-γ coactivator PPAR-γ coactivator-1α (PGC-1α), and corepressors and binds to the PPAR-γ response element (PPRE) in the promoter region of selected genes. Its pattern of response involves genes active in the control of metabolic events and oxidative stress control [6,12,13]. In previous studies, we and others have contributed to characterizing the effects of PPAR-γ agonists in OPs in culture, pointing out the induction of genes involved in ROS control and the capability to promote OL differentiation [12,14,15]. More recently, we focused our attention on the role of PPAR-γ in the control of mitochondrial function and on the link between this latter and OP differentiation [6].

**Figure 1:**
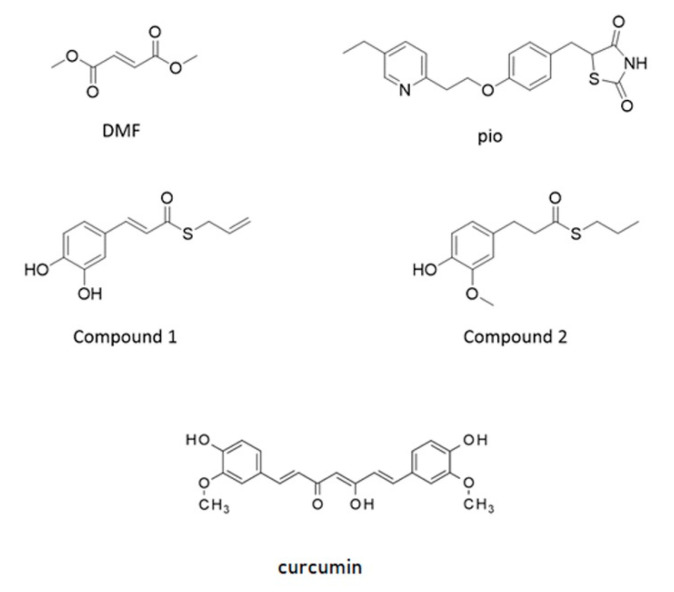
Structure of molecules used as agents activating nuclear factor-erythroid 2-related factor (NRF2) and peroxisome proliferator-activated receptor gamma (PPAR-γ). Structure of dimethyl-fumarate (DMF), pioglitazone (pio), curcumin (Curc), and compounds synthesized by combining the hydroxycinnamoyl motif derived from curcumin and the allyl mercaptan moiety of garlic organosulfur compounds (Compound 1 and Compound 2). Compound 1 bears a catechol moiety and an α,β-unsaturated carbonyl group, both conferring electrophilic features; compound 2 lacks these moieties.

NRF2 is capable of amplifying the self-defense against oxidative and xenobiotic insults. At the resting state, the basal activity of NRF2 is downregulated by chronic degradation induced by Kelch-like ECH-associated protein 1 (Keap1). Following the oxidation of cysteine residues on Keap1 by ROS, xenobiotics, and electrophiles, NRF2 is released from Keap1 binding and moves to the nucleus. As a dimer with small musculoaponeurotic fibrosarcoma (maf) protein, NRF2 interacts with the antioxidant responsive element (ARE) sequences on the promoter of several genes involved in ROS and xenobiotic defense and initiates the so-called phase II antioxidant detoxifying defense [16,17,18]. Due to its neuroprotective, antioxidative, and anti-inflammatory capacities, NRF2 is considered as a target candidate for therapeutic interventions in demyelinating and neurodegenerative diseases [19,20,21]. Besides the very well-known role of NRF2 in the orchestration of the cell response to stress, recent evidence is accumulating on its capability to control the efficiency of the mitochondrial compartment [19,22,23]. Worthy of note, PPAR-γ and NRF2 share antioxidant and mitochondria protective functions, and both of them have been identified as targets of pharmacological approaches in therapies aimed at curing type 2 diabetes and relapsing-remitting multiple sclerosis, respectively [11]. To point out specificities and similarities in the effects of the two pathways and, as a future perspective, to consider these transcription factors as targets in a combined therapy aimed at treating myelin diseases, we analyzed two drugs currently used in clinical practice: pioglitazone, a PPAR-γ agonist belonging to the family of thiazolidinediones, and the NRF2 inducer dimethyl fumarate. We also tested, in our purified culture of OPs, two molecules, compounds 1 and 2, previously shown to induce the NRF2/ARE pathway in the SH-SY5Y neuroblastoma cell line [24,25]. They were selected from a small set of compounds synthesized by combining the hydroxycinnamoyl motif derived from curcumin and the allyl mercaptan moiety of garlic organosulfur compounds. Compound 1 bears electrophilic features previously shown to be responsible for the activation of the NRF2. Conversely, compound 2, lacking these features, is not able to induce the NRF2 pathway (Figure 1) [24,25]. Curcumin was also tested as the parent compound.

## 2. Results

### 2.1. Only Dimethyl-Fumarate (DMF) Causes a Transient Mitochondrial Inner-Membrane Potential (mMP) Depolarization Due to Glutathione (GSH) Depletion and Mitochondrial Superoxide Production

Pioglitazone (Pio) and DMF concentrations were chosen based on previous [12] and preliminary studies, to avoid significant effects on the cell viability.

**Figure 2:**
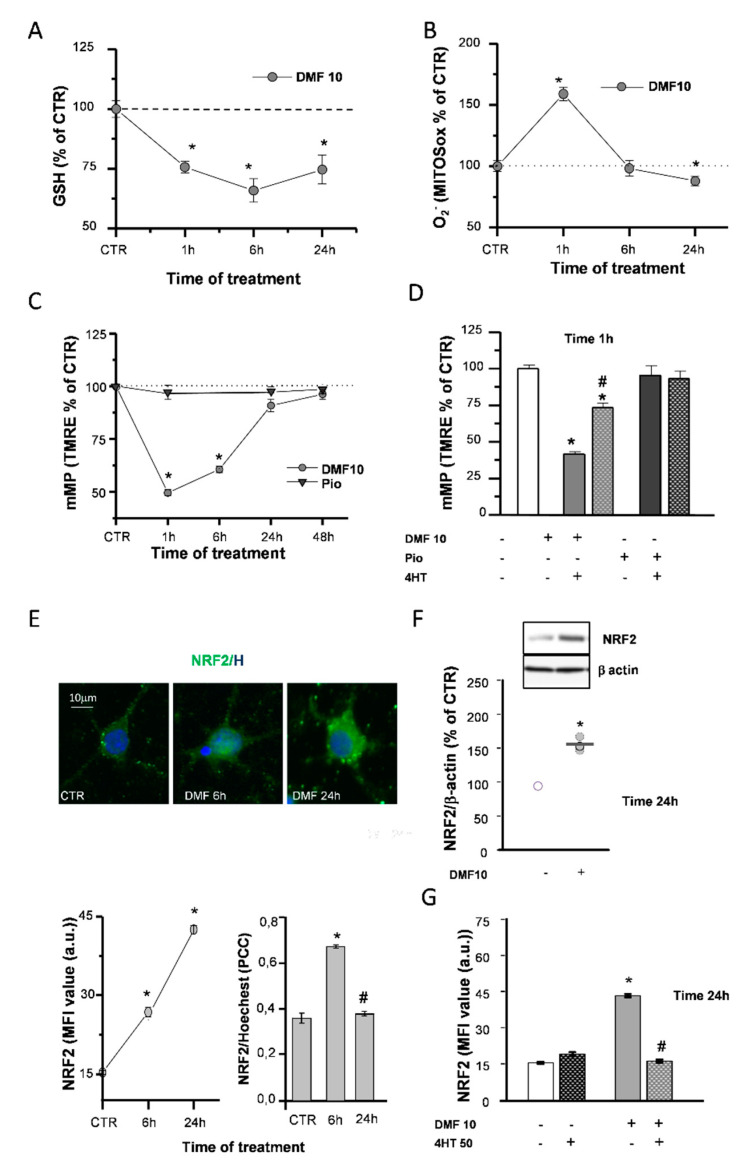
DMF causes early effects on the mitochondria and NRF2 induction. Glutathione (GSH) levels were measured by a luminescence assay kit to evaluate the effects of 10-µM DMF at different time points (1, 6, and 24 h) (**A**). Untreated (CTR) is taken as 100%. Experimental points are mean ± SEM of *n* = 4 experiments (* *p* < 0.05 vs. CTR). Fluorescence intensities of single cells loaded with the mitochondrial superoxide sensitive probe (MitoSOX) were measured at different time points (1, 6, and 24 h) during 10-µM DMF treatments (**B**). Intensity values were normalized to the untreated (CTR) value and shown as mean ± SEM from *n* = 317-507 cells (* *p* < 0.05 vs. CTR). Tetramethylrhodamine-ethyl ester (TMRE) fluorescence intensities of single mitochondria were measured at different time points (1, 6, 24, and 48 h) during treatments with 1-µM pio or with DMF 10µM (**C**). Data are expressed as mean ± SEM from *n* = 120–1627 mitochondria (* *p* < 0.05 vs. CTR). Oligodendrocyte progenitor (OP) cultures were treated for 1 h with 10-µM DMF or 1-µM pio alone or with 50-µM 4-hydroxy-TEMPO (4HT) pretreatment. TMRE fluorescence intensity was measured. CTR is taken as 100%. Data are expressed as mean ± SEM from *n* = 45–182 mitochondria (* *p* < 0.05 vs. CTR and # *p* < 0.05 vs. DMF) (**D**). OP cultures treated with 10-µM DMF for different time points (6 and 24 h) were immunostained for NRF2 and Hoechst (**E**). The panel above shows micrographs of exemplifying cells in different conditions. The panel below shows the mean fluorescence intensities (MFI) of NRF2 and the colocalization of NRF2 and Hoechst evaluated by Pearson’s correlation coefficients (PCC). PCC = 1 corresponds to maximal colocalization. Data are mean ± SEM of *n* = 250–300 cells (3 independent experiments; * *p* < 0.05 vs. CTR and # *p* < 0.05 vs. 6 h). Western blot analysis of OP cultures treated for 24 h with 10-µM DMF are shown (**F**). Representative bands of β-actin and NRF2 are shown above, and densitometric analysis is shown below (data are expressed as mean ± SEM from *n* = 3 experiments; * *p* < 0.05 vs. CTR). OP cultures treated with 10-µM DMF alone or with 50-µM 4HT pretreatment were immunostained for NRF2. MFI ± SEM from *n* = 250–300 cells are shown (* *p* < 0.05 vs. CTR and # *p* < 0.001 vs. DMF) (**G**).

DMF is known to react with glutathione (GSH) rapidly and to provoke a quick depletion of its reduced form [26,27], which could affect the mitochondrial respiratory chain efficiency. After 1h of DMF treatment, GSH levels decreased in a concentration-dependent manner (Figure S1A). Furthermore, in the continuous presence of 10-µM DMF, GSH levels were still reduced after 6h, while they recovered after 24h, although not fully recovering to the control (CTR) levels (Figure 2A).

Accordingly, such an early GSH decrease was paralleled by mitochondrial superoxide production, as evidenced by live fluorescence experiments with the dye mitochondrial superoxide (MitoSOX) (Figure 2B). When the mitochondrial inner-membrane potential (mMP) was measured in live fluorescence experiments with the potentiometric dye tetramethylrhodamine-ethyl ester (TMRE), DMF caused an early (1 h) mMP depolarization that recovered back to the CTR in the continuous presence of the agent (Figure 2C and Figure S1B). As a confirmation of the involvement of ROS in the DMF-induced mMP depolarization, this latter was prevented by 4-hydroxy-TEMPO (4HT), an antioxidant agent mimicking superoxide dismutase activity (Figure 2D). On the other hand, the activation of the PPAR-γ pathway with pio did not cause an early (1 h) mMP depolarization, and the 4HT co-application did not affect it (Figure 2C,D).

### 2.2. DMF Induces NRF2 and its Nuclear Translocation

DMF was described in a variety of cell types to exert its effects by inducing the NRF2/ARE axis of transcriptional activation. Immunofluorescence (IF) and Western blotting (WB) were used to evaluate the capability of DMF to activate NRF2 in OPs. DMF treatment induced an increase in NRF2 levels, at both 6 and 24 h, as well as NRF2 nuclear translocation at 6 h (Figure 2E and Figure S1C). WB experiments confirmed the increase of NRF2 levels at 24 h (Figure 2F). Worthy of note, when coapplied with DMF, 4HT prevented NRF2 induction (Figure 2G), in keeping with the contribution of the early mitochondrial effects (i.e., mMP depolarization and superoxide production) in the mechanism responsible for DMF-mediated NRF2 induction.

### 2.3. DMF and Pio Induce the Expression of PGC-1α and Increase Mitochondrial Biogenesis

**Figure 3:**
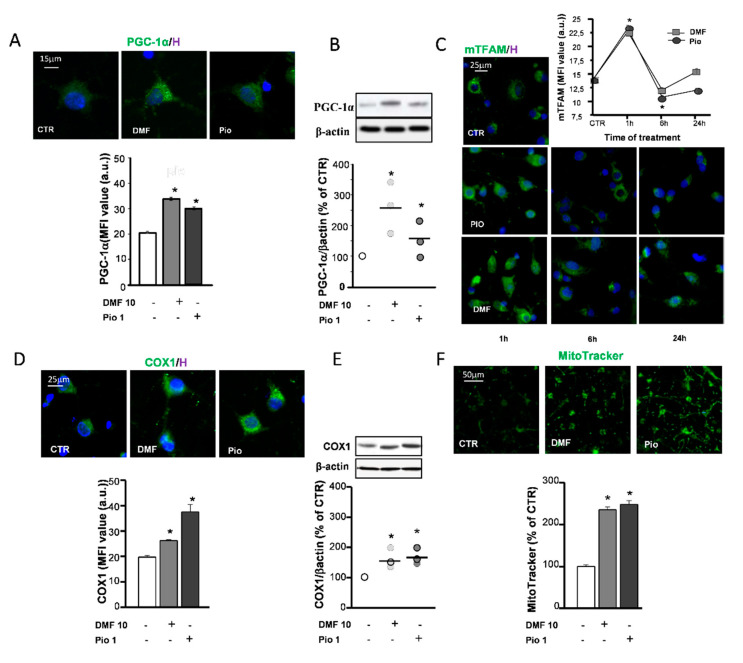
DMF and pio induce mitochondrial biogenesis. OP cultures treated for 24 h with 10-µM DMF or 1-µM pio were processed for IF experiments with PPAR-γ coactivator-1α (PGC-1α) (**A**). The micrographs show PGC-1α (green) and Hoechst 33258 nuclear fluorochrome (blue, H). The bar graph shows MFI analysis (MFI ± SEM from *n* = 250–300; * *p* < 0.05 vs. CTR). WB analysis of PGC-1α expression is shown (**B**). Representative bands of β-actin and PGC-1α are shown above; densitometric analysis is shown as mean and individual data points in the graph below (*n* = 3; *p < 0.05 vs. CTR). OP cultures treated for different time points (1, 6, and 24 h) with 10-µM DMF or 1-µM pio were immunostained for transcription factor A (TFAM) (**C**). The micrographs show TFAM (green) and Hoechst 33258 (blue, H). The graph shows the MFI analysis (*n* = 250–300 cells; * *p* < 0.005 vs. CTR). OP cultures treated with 10-µM DMF or 1-µM pio were processed for IF experiments with complex IV core protein (COX-1) (**D**). The micrographs show COX-1 (green) and Hoechst 33258 (blue, **H**). The bar graph shows the MFI analysis (*n* = 250–300 cells; * *p* <0.05 vs. CTR). WB analysis of COX-1 expression is shown (**E**). Representative bands of β-actin and PGC-1α are shown above; densitometric analysis is shown as mean and individual data points in the graph below (*n* = 3; * *p* < 0.05 vs. CTR). OP cultures were treated with 10-µM DMF or 1-µM pio and then loaded with the mitochondrial probe MitoTracker Green (**F**). MitoTracker fluorescence intensities were measured from single cells, and mean ± SEM are shown in the bar graph. CTR is taken as 100% (*n* = 308–460 cells; * *p* < 0.05 vs. CTR).

The replacement of mitochondrial components targeted by oxidative stress and increase of mitochondrial mass to comply with the rise of energetic and metabolic needs are accomplished by mitochondrial biogenesis. One of the main factors controlling mitochondrial biogenesis is the PPAR-γ coactivator-1α (PGC-1α), known to act as a coactivator of a variety of transcription factors—among these, PPAR-γ. The capability of pio to increase the expression of PGC-1α has already been documented in previous studies [28,29]. Herein, we confirmed the effect of pio and highlighted the activity of DMF in upregulating the PGC-1α protein expression, as shown by both mean fluorescence intensities (MFI) analysis of IF experiments and WB densitometric analysis (Figure 3A,B). Another factor among those involved in the orchestration of mitochondrial biogenesis is the mitochondrial transcription factor A (TFAM). Coded in the nuclear genome, the TFAM protein, once transcribed and transported in the mitochondrial matrix, participates to the induction of mitochondrial gene expression. Both pio and DMF were capable of upregulating the TFAM expression already after 1h treatment. At later times (6 and 24 h), the TFAM levels declined (Figure 3C), probably due to the rapid inclusion in mitochondrial nucleoids [30]. Two approaches were utilized to ascertain whether mitochondrial biogenesis occurred as a result of the DMF and pio treatment: the upregulation of the expression of the complex IV core protein (COX1) and the measurement of fluorescence emitted by the mitochondrial dye MitoTracker Green. The COX1 expression was increased by both DMF and pio, as highlighted by the IF and WB experiments. The fluorescence intensity analysis of MitoTracker-loaded cells allowed to point out the increase of the mitochondrial mass in cells treated with the agents (Figure 3D–F).

### 2.4. DMF and Pio Differently Regulate the Expression of the Antioxidative Enzymes Mn Superoxide Dismutase (MnSOD) and Heme Oxygenase 1 (HO-1)

**Figure 4:**
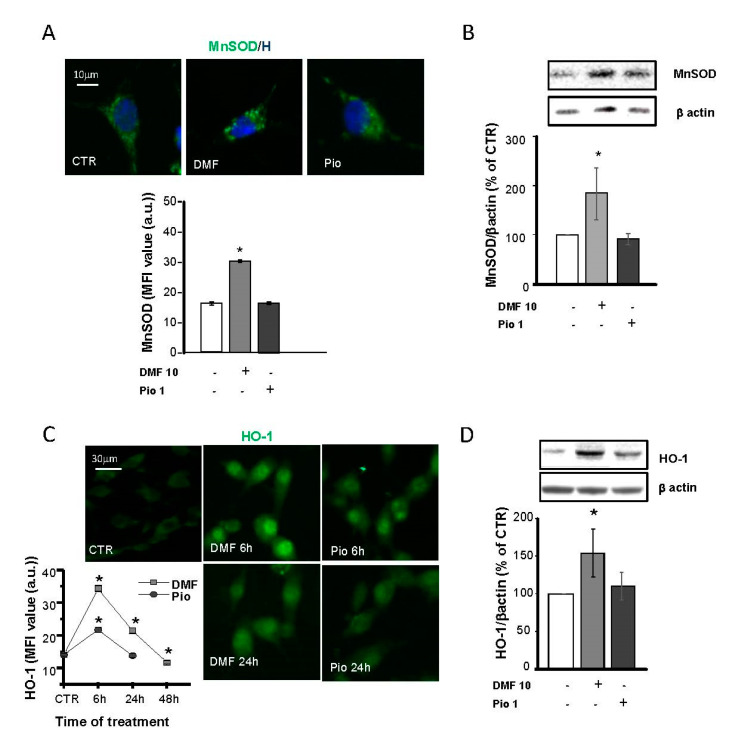
DMF and pio differently regulate the antioxidant enzymes heme oxygenase 1 (HO-1) and MnSOD. OP cultures treated for 24 h (unless indicated) with 10-µM DMF or 1-µM pio were immunostained for MnSOD (**A**). The micrographs show MnSOD (green) and Hoechst 33258 (blue, H). The bar graph shows MFI analysis of the CTR and treated cells (*n* = 250–300 cells; * *p* < 0.05 vs. CTR). WB analysis of MnSOD expression is shown (**B)**. Representative bands of MnSOD and β-actin are shown above. Mean and individual data points from the densitometric analysis are shown in the graph below (*n* = 3; * *p* < 0.05 vs. CTR). OP cultures were treated with 10-µM DMF or 1-µM pio and then processed for IF experiments for HO-1 (**C**). The micrographs show HO-1 (green). The bar graph shows MFI (*n* = 250-300 cells; * *p* < 0.05 vs. CTR). WB analysis of HO-1 expression is shown (**D**). Representative bands of β-actin and HO-1 are shown above. Mean and individual data points from the densitometric analysis are shown in the graph below (*n* = 3; * *p* < 0.05 vs. CTR).

Protection against oxidative stress is essential for the successful remyelination by OPs, especially considering the highly inflamed environment and oxidative stress-taking place at sites of demyelination. In previous studies, MnSOD, Cu, ZnSOD, and catalase were shown to be highly expressed in the same culture conditions used in this study [12]. Moreover, PPAR-γ was shown to upregulate Cu, ZnSOD, and catalase but not MnSOD. To depict the specificities and similarities in the antioxidant capabilities of the NRF2 and PPAR-γ pathways, we considered MnSOD, a SOD isoform localized in the mitochondrial matrix, and heme oxygenase 1 (HO-1), which was shown to act as a triggering factor of the phase II antioxidant response initiated by NRF2. In IF experiments, MnSOD showed a punctate distribution compatible with its mitochondrial localization. As expected, pio was unable to increase the MnSOD expression, as demonstrated in both IF and WB experiments (Figure 4A,B).

On the contrary, NRF2 activation by DMF caused a significant increase of MnSOD expression, in keeping with what was already shown in other cell types [31,32,33]. IF and WB analyses were used to point out differences in DMF- and pio-induced changes in the HO-1 expression. DMF induced a much more significant and long-lasting increase of HO-1 compared to pio: the induction peaked at 6h for both agents, but at 24h in pio-treated OPs, the level of HO-1 was comparable to the CTR (Figure 4C,D). In IF experiments, the HO-1 fluorescence signal was clustered in the nuclear space, in keeping with previous findings and with the hypothesized role in the regulation of the phase II gene transcription [34,35].

### 2.5. Different Mechanisms of Protection by DMF and Pio from Tumor Necrosis Factor-Alpha (TNF-α) and Rotenone-Induced Mitochondrial Depolarization

**Figure 5:**
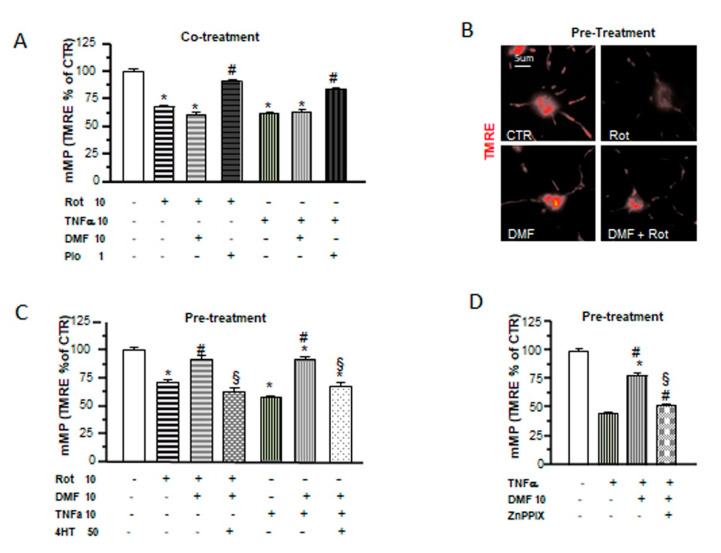
DMF and pio differently protect from inflammatory and mitochondrial insults. OP cultures treated for 24 h with 10-nM rotenone (Rot) or 10-ng/mL TNF-α alone or in the presence of 10-µM DMF or 1-µM pio were loaded with the potentiometric probe TMRE. TMRE fluorescence intensities from mitochondria were measured and shown in the bar graph (**A**). CTR is taken as 100% (mean ± SEM, *n* = 80-246 mitochondria; * *p* < 0.05 vs. CTR and # *p* < 0.05 vs. stress). Examples of OPs loaded with TMRE under different culture conditions are shown (**B)**. OP cultures were treated for 24 h with 10-nM rotenone (Rot) or 10-ng/mL TNF-α. Some Rot-treated cultures were pretreated with 10-µM DMF for 24 h alone or in the presence of a 4HT pretreatment. Fluorescence from TMRE-loaded mitochondria was measured after 48 h of treatment and shown in the bar graph (**C**). CTR is taken as 100%. Mean ± SEM; *n* = 40–260 mitochondria (* *p* < 0.05 vs. CTR, # *p* < 0.05 vs. stress, and § *p* < 0.05 vs. DMF+stress). As in (**C**), OP cultures treated with TNF-α for 24 h were pretreated with DMF alone or in the presence of 2-µM zinc protoorphirin-9 (ZnPPIX). Fluorescence from TMRE-loaded mitochondria was measured and shown in the bar graph (**D**) (mean ± SEM; *n* = 60–120 mitochondria; * *p* < 0.05 vs. CTR, # *p* < 0.05 vs. stress, and § *p* < 0.05 vs. DMF+stress).

In a recent study, we demonstrated the capability of pio to protect OPs from the effects of the proinflammatory cytokine TNF-α and the mitochondrial toxin rotenone on mitochondrial depolarization and to slow down OP differentiation. Moreover, pio was able to recover mMP from the toxic effect of TNF-α not only when coapplied but, also, when added 24h after the toxic agents [28]. In this study, we tested DMF in the same experimental design and compared its effects with those formerly obtained with pio. When added together with the toxins, DMF was incapable of preventing mMP depolarization (Figure 5A). However, a 24-h pretreatment with the electrophile was sufficient to avoid mMP depolarization (Figure 5B,C). The protective effect of DMF on mMP depolarization induced by TNF-α was lost in the presence of the antioxidant 4HT or the HO-1 inhibitor Zn protoporphyrin IX (Figure 5C,D), suggesting, once more, a functional link between the early mitochondrial effects (i.e., mMP depolarization and superoxide production) induced by DMF and its protective effect and highlighting the important role played by HO-1 in DMF-induced mitochondrial protection.

### 2.6. DMF and Pio Differently Regulate OP Differentiation

**Figure 6:**
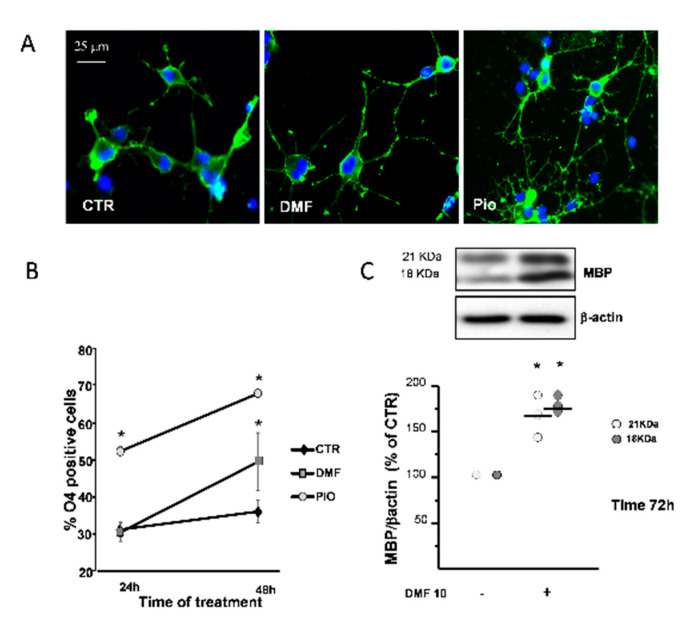
DMF and pio differently promote OP differentiation. OP cultures treated for 24 or 48 h with 10-µM DMF or 1-µM pio were immunostained for O_4_ (**A**). The % of O_4_-positive cells was calculated, and mean ± SEM are shown in the graph (**B**) (*n* = 5 to 6 experiments; * *p* < 0.05 vs. CTR). OP cultures were treated for 72 h with DMF 10 and then processed for WB of the myelin basic protein (MBP) and β-actin (**C**). Representative bands of β-actin and MBP at the two different molecular weights (18 and 21 kDa) are shown above. Mean ± individual data points from the densitometric analysis are shown in the bar graph below (*n* = 3; * *p* < 0.05 vs. CTR).

PPAR-γ is known to promote OL differentiation [12,36]. The O_4_ monoclonal antibody, known to bind to membrane sulphatides, is currently used to identify pre-OLs, the first stage of post-mitotic OLs. In complete agreement with previous studies, pio acted as a very efficient OL pro-differentiation factor, since it nearly doubled the fraction of O_4_^+^ cells at 24h and 48h treatments, as compared to the CTR. DMF caused a significantly slower pro-differentiation effect compared to pio, as the number of O_4_^+^ cells was unchanged after 24h and increased only after 48h treatment (Figure 6B). DMF also had a weaker effect in inducing changes in the cell morphology, which are known to accompany OL maturation, as compared to pio (Figure 6A).

As a confirmation on the capacity to promote differentiation, after three days of treatment, DMF induced the increase of myelin basic protein (MBP), a protein characteristic of later phases of the OL lineage (Figure 6C).

### 2.7. Effects of Hybrids on the NRF2/ARE Axis

A small set of molecules has been previously synthesized by combining the functional moieties of curcumin and diallyl sulfide: curcumin is known to activate the NRF2 and PPAR-γ pathways [25,37,38], and diallyl sulfide is known to induce the expression of antioxidant phase II enzymes by activating the NRF2 pathway [24,39] (Figure 1). Among these hybrids, compound 1 was found to be one of the most active in modulating the activation of NRF2 pathway [24,25].

**Figure 7:**
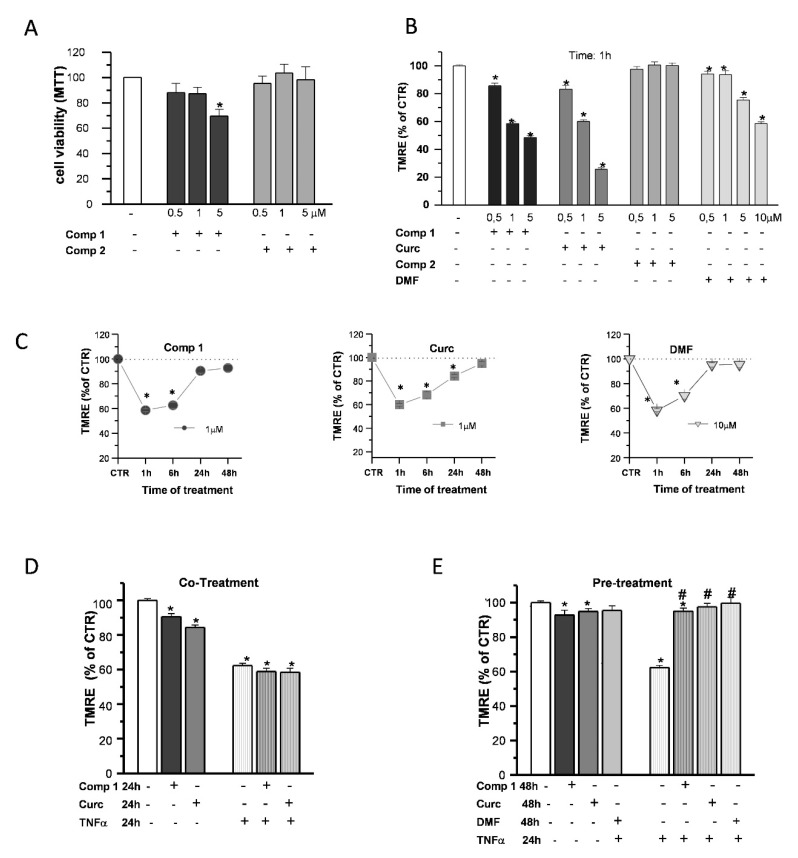
Effects of hybrids on the mitochondria. OP cultures were treated for 24 h with different concentrations (0.5, 1, and 5 µM) of the synthetic compounds, and cell viability was determined by 3-(4,5-dimethyl thiazol-2-y1)-2,5-diphenyl tetrazolium bromide (MTT) assay (**A**). TMRE fluorescence of single mitochondria were measured after 1-h treatments at different concentrations (0.5, 1, and 5 µM) of compound 1 (comp 1), curcumin (Curc), DMF, or compound 2 (comp 2) and were shown in the histogram (**B**). TMRE fluorescence of single mitochondria were also measured at different time points (1, 6, 24, and 48 h) during treatments at the chosen concentrations of 1 µM (comp 1 and Curc) and 10 µM (DMF) and shown in the scatter graphs (**C**). Experimental points are mean ± SEM from *n* = 80–540 mitochondria. OP cultures were treated for 24 h with 10-ng/mL TNF-α alone or in the presence of comp 1 or Curc. Fluorescence from TMRE-loaded mitochondria was measured and was shown in the bar graph. CTR is taken as 100% (mean ± SEM; *n* = 60–460 mitochondria; * *p* < 0.05 vs. CTR) (**D**). OP cultures treated for 24 h with 10-ng/mL TNF-α were pretreated or not for 24 h with DMF, comp 1, or Curc. After 48-h treatment, the fluorescence from TMRE-loaded mitochondria was measured and was shown in the bar graph. CTR is taken as 100% (mean ± SEM; *n* = 60–460 mitochondria; * *p* < 0.05 vs. CTR and # *p* < 0.05 vs. TNF) (**E**).

To gain some insight on the effects of compound 1 in OP cultures, the same doses of compound 1, compound 2, curcumin, and DMF were tested for comparison. Based on the previous data obtained on SH-SY5Y cells [24,25], we performed the experiments with increasing concentrations (0.5, 1, and 5 µM) of the compounds. The cell viability of OPs exposed to compounds 1 and 2 was determined by the 3-(4,5-dimethyl thiazol-2-y1)-2,5-diphenyl tetrazolium bromide (MTT) assay (Figure 7A). Only in the case of compound 1, at 5-μM concentration, cell viability was reduced by about 20%, consistent with the previous data in SH-SY5Y cells [24,25].

When mMP was measured in live fluorescence experiments with the potentiometric dye TMRE, compound 1 and curcumin, but not compound 2, caused an early (1-h) mMP depolarization that recovered back to the CTR in the continuous presence of the agents (Figure 7B). Compound 1 and curcumin showed a higher potency compared to DMF, since a concentration of 1 µM was sufficient to induce a decrease of TMRE fluorescence intensity comparable to that of DMF at the dose of 10 μM: after 1-h treatments, about 43%, 40%, and 42% with respect to the CTR, respectively. In the presence of all the agents, mMP returned to the control levels in 48 h (Figure 7C).

Finally, we studied the ability of compound 1 to protect OPs from the effects of the proinflammatory cytokine TNF-α on mitochondrial depolarization, selecting 1 µM as the optimal concentration based on the cell viability and mMP results. Unlike pio (Figure 5A), compound 1 and curcumin were incapable to prevent mMP depolarization when coapplied with the toxins. At the same time, as DMF, a 24-h pretreatment was necessary to inhibit the TNF-α effect (Figure 7D,E).

## 3. Discussion

To study the NRF2 and PPAR-γ pathways in OL cultures, we evaluated the effects induced by agents known to activate such transcription factors on some representative events, such as early effects on the mitochondria, NRF2 levels, antioxidant repertoire, mitochondrial biogenesis, protection against inflammatory and mitochondrial insults, and OP differentiation.

### 3.1. DMF-Induced Mitochondrial Early Events and NRF2 Induction

Electrophiles such as DMF are known to cause GSH depletion. In keeping with this notion, we found a sudden decline of GSH paralleled by a fast raise of mitochondrial superoxide production. Worthy of note, the mitochondrial membrane potential (mMP) of cells challenged with different doses of DMF, but not of pio, showed an early (1-h) depolarization, followed by a slow recovery to the control levels. Due to the oxidation of GSH to GSSG by DMF, we can envisage the following events: oxidation of Krebs cycle enzymes and respiratory chain complexes causing a slowdown of the electron flux and, in turn, mMP depolarization, gluthationization of complex I, and further rise of superoxide and mMP depolarization [40]. The cause/effect relationship between mitochondrial ROS production and mMP depolarization is compatible with the abrogation of depolarization in the presence of the antioxidant 4-hydroxy-TEMPO (4HT).

Our unprecedented observations on superoxide production and mMP depolarization, besides providing new insights for the comprehension of electrophile toxicity, might add information useful to better understand the mechanism of the activation of NRF2 by electrophiles.

We confirmed the DMF capability to activate the NRF2 pathway also in OPs. Moreover, NRF2 induction was sensitive to the presence of 4HT, as the early effect of DMF on mMP. We can envisage the unprecedented hypothesis that the early mitochondrial effect by DMF plays a significant role in NRF2 induction. For this hypothesis to hold true, it remains to be ascertained that 4HT is incapable of overcoming the alkylation of thiol groups on Keap1 directly.

### 3.2. DMF- and Pio-Induced PGC-1α and Mitochondrial Biogenesis

A large body of evidence supports the importance of a correct mitochondrial function for OP differentiation and myelination. The replacement of damaged mitochondria or their damaged constituents is comprised in the phenomenon called mitochondrial biogenesis. This event is a complex and highly regulated process in which the proper coordination of the expression of nuclear and mitochondrial genes, the transport of new proteins in the mitochondria, and their assembly in their final locations must be optimized. To decipher whether the activation of PPAR-γ and NRF2 affect mitochondrial biogenesis in OPs, we chose to evaluate the expression of the coactivator PGC-1α and the transcription factor TFAM. The former is capable of orchestrating mitochondrial biogenesis by acting as the coactivator of several transcription factors; the latter is one of the leading transcription factors implicated in the induction and coordination of the expression of the mitochondrial genome. As shown in the IF analysis and WB experiments, both agents induced a rise in the expression of PGC-1α and TFAM. Although the PGC-1α induction by PPAR-γ activation has been described in the literature, in OPs, the PGC-1α induction by the NRF2 activator DMF was even more efficient. A complex and apparently contradictory picture on PGC-1α induction by NRF2 activation comes from studies in cell types different from OPs. Examples are, on the one hand, the increase of PGC-1α and mitochondrial mass in fibroblasts by the NRF2 inducer sulforaphane, and, on the other hand, the lower PGC-1α levels in liver cells overexpressing NRF2 following knocking down Keap1 [41,42]. Such an apparent contradiction might be reconciled considering the opposite effect of NRF2 acute activation by the agonist vs. the long-lasting NRF2 activation by Keap1-knockdown.

PPAR-γ and NRF2 activation by DMF and pio, respectively, resulted in a fast upregulation of TFAM. The rise of TFAM MFI peaked at 1h from the beginning of the treatment and, at 6h, was not any longer detectable. Such a fast TFAM expression is not unprecedented, and, most probably, the disappearance of TFAM immunoreactivity did not correspond to a protein loss but, rather, to the incorporation of TFAM in the multimolecular complexes, including also single mitochondrial DNA known as nucleoids [30,43].

To confirm that the increased expression of PGC-1α and TFAM was responsible for the actual rise of the mitochondrial components, we utilized two strategies: biochemical evaluation of the expression of COX1 and fluorescence evaluation of the mitochondrial mass with the mitochondrial dye MitoTracker Green. Both agents were capable per se to increase the expression of COX1 and the fluorescence signal by MitoTracker.

These data support the capability of both transcription factors (NRF2 and PPAR-γ) to sustain mitochondrial biogenesis. The promotion of mitochondrial biogenesis would add further therapeutic potential, considering the need for mitochondrial efficiency for differentiation to occur and the highly inflammatory and oxidative environment characteristics of sites of demyelination, which are detrimental for mitochondria.

### 3.3. Different Effects of NRF2- and PPAR-γ- Activation on the Antioxidant Enzymes MnSOD and HO-1

The repertoire of antioxidant systems governed by NRF2 and PPAR-γ has been described in previous studies. PPAR-γ induction was described to cause the upregulation of the catalase of Cu, ZnSOD but not MnSOD [44,45,46]. On the other hand, NRF2 activation is known to build up a vast repertoire of enzymes involved in the defense against oxidants, among which are HO-1 and MnSOD (reviewed in [47]).

To depict the similarities and specificities of DMF and pio (NRF2 and PPAR-γ), we focused our attention on HO-1 and MnSOD. The former is one of the most important antioxidant enzymes among those upregulated by NRF2. It degrades heme in the two antioxidant molecules biliverdin and CO but also participates in the regulation of the induction of the whole repertoire of the NRF2-induced response. Both agents were capable of increasing the HO-1 levels but with the NRF2 inducer showing a more significant effect. In keeping with the previous observations and its role in the NFR2 response orchestration, HO-1 quickly moved to the nucleus.

MnSOD is found in the mitochondrial matrix, where it plays a role in detoxification from superoxide radicals produced mainly by complexes I and III of the respiratory chain. In addition to its protective role, it is also involved in ROS-mediated signaling through the production of hydrogen peroxide, which is then released in the cytoplasm [31,48,49]. Is hydrogen peroxide produced by MnSOD capable of contributing to NRF2 induction by the oxidation of thiols on Keap1, as our data on the inhibitory effect of 4HT would suggest? In keeping with previous observations in cells other than OPs, DMF induced the expression of MnSOD. In this and earlier studies, pio was shown not to affect the MnSOD levels. At this regard, the literature is contradictory. In-line with our results, two PPRE sequences were computationally predicted in the human MnSOD promoter, and their activation by the endogenous PPAR-γ agonist 15d-PGJ2 caused MnSOD gene repression. The lack of induction of MnSOD could be a specific orientation of PPAR-γ signaling, aimed to induce differentiation and mitochondrial oxidation and to inhibit oncogenic proliferation and glycolytic flux [31,50]. However, PPAR-γ induced MnSOD in other studies/cell types, as has been observed, e.g., in astrocytes, 3T3-L1 preadipocyte cells, and C2C12 skeletal muscle cells [51,52,53,54].

### 3.4. Different Effects of PPAR-γ and NRF2 Activation on Mitochondrial Protection

In a previous study, we specifically evaluated the capability of pio, as a PPAR-γ agonist, to overcome the effect of the proinflammatory cytokine TNF-α and the mitochondrial toxin rotenone on OP differentiation. In parallel, we evaluated the effects on some mitochondrial properties, such as the decrease of membrane potential, superoxide production, and oscillatory Ca^2+^ signals, which we have previously described as depending on mitochondrial function. Pio was capable of rescuing OP differentiation and of counteracting the effect of TNF-α on the above parameters. Moreover, and of interest in a therapeutic perspective, pio was also active when administered after the effect of TNF-α occurred. Herein, we applied the same experimental protocol formerly used for pio, with the intent of comparing the protective effect of PPAR-γ- with NRF2-activation on mitochondria stressors (i.e., TNF-α and rotenone). Interestingly, we observed that a 24-h preapplication of the NRF2 activator DMF was required to obtain protection against the cytokine. Such a distinct pattern of protection can be accounted for considering the events that discriminate NRF2 and PPAR-γ activation: the initial effects on the mitochondria and the effects on HO-1. This latter is of great importance for the protective mechanism induced by NRF2, since with the HO-1 inhibitor Zn-protoporphyrin, the protection by DMF was lost. However, the stronger action of DMF on HO-1 induction would cause a more efficient protection. More probably, the early effects on mMP, which is blocked by 4HT, is the cause of the different kinetics of protection.

### 3.5. Different Effects of NRF2- and PPAR-γ-Activation on OP Differentiation

Any therapeutic intervention aimed at recovering from myelin loss must promote the differentiation of the cells responsible for reforming the myelin sheaths. A robust body of evidence supports the capability of PPAR-γ agonists to act as pro-differentiation factors and to push OP differentiation toward myelin-forming cells. More controversial is the effect of NRF2 on differentiation. In bone tissue, most observations support an inhibitory role of NRF2 in osteoblast/osteoclast differentiation [55]. On the other hand, NRF2 promotes adipocyte differentiation by a dual mechanism: the induction of the transient expression of CCAAT/enhancer-binding protein β (CEBPβ) and the upregulation of PPAR-γ through binding to the ARE sequence in the promoter regions of those genes [56].

In our experimental setting, PPAR-γ and NRF2 activation differed remarkably in their ability to induce OP differentiation. In-line with its known capability to induce cell differentiation, pio exerted a powerful and fast effect on OP differentiation at all the stages studied. At odds, earlier phases of OP differentiation, described by O_4_, were only slightly affected by DMF after 48 h of culturing. On the other hand, MBP induction by DMF proves the capability of this agent to promote OL differentiation at later times.

### 3.6. Effects of Hybrids on the NRF2/ARE Axis

We also analyzed other compounds able to activate the NRF2 and PPAR-γ pathways in search of those competent to protect from mitochondrial stressors at lower concentrations. Curcumin is known to induce both the PPAR-γ and NRF2 pathways. As pio, curcumin induces cell differentiation and protection from TNF-γ damage through activation of the PPAR-γ pathway in OPs [38]. On the other hand, curcumin induces phase II enzymes and protects from oxidative and mitochondrial stress by activating NRF2 in different cell types [57,58]. In this study, we demonstrate that, in OPs, the effect of curcumin on mMP is similar to the DMF effect, suggesting an electrophile-type effect. We also tested two compounds synthesized by combining the functional moieties of curcumin and diallyl sulfide [24,39].

It is known that the hydroxycinnamoyl motif regulates several pathways related to neurodegenerative diseases [24,59] and that the allyl mercaptan moieties counteract oxidative stress through the induction of antioxidant enzyme expression. Compound 1 combines the electrophilic α,β-unsaturated carbonyl group (Michael acceptor functionality) with the pro-electrophilic catechol moiety, which is proved able to activate the NRF2-driven pathway. In contrast, compound 2 is inactive in this respect because of lacking both (pro) electrophilic functionalities. The mechanism at the basis of the effects exerted by compound 1 was hypothesized to be dependent on direct interactions with Keap1 by possibly modifying the sulfhydryl groups of cysteine residues on Keap1 and inhibiting the Keap1-NRF2 protein-protein interaction, which drives the proteasome-dependent NRF2 degradation. A proof of the hypothesis of an electrophile-based modulation of the NRF2 pathway, as discussed by Basagni et al. [60], is the lack of efficacy in activating NRF2 observed for compound 2, which, lacking (pro)electrophilic features, is not able to form a covalent adduct with the cysteine residues of Keap1.

In a recent study, we presented data on the capability of compound 1 to induce NRF2 in the human SH-SY5Y neuroblastoma cell line and to promote the endogenous upregulation of NRF2-dependent defensive genes such as NQO1 and HO-1 [24,25].

In this study, we demonstrate that, in OPs, the effects of compound 1 on the mitochondrial functions are like those of DMF. Compound 1, differently from compound 2, was able to induce early mitochondrial depolarization and to protect against TNF-α-induced mitochondrial damage like DMF but at a lower concentration.

### 3.7. Concluding Remarks

This study aimed to gather in a single research relevant information on two transduction pathways, PPAR-γ and NRF2, targeted by drugs also approved for demyelination diseases, such as multiple sclerosis. Evidence is accumulating on the crosstalk between such pathways that can envisage common goals and possible synergisms to be exploited in a therapeutic perspective.

The intriguing hypothesis of crosstalk between the NRF2 and PPAR-γ pathways, aimed at reinforcing the cell response against oxidative stress and supporting the mitochondrial function, is in keeping with the presence of ARE and PPRE sequences in the promoter regions of both transcription factors [61].

The crosstalk between the two transcription pathways can be of great benefit for the efficacy of a therapeutic strategy based on the co-stimulation of the two. However, it makes it essential to fully comprehend their reciprocal relationship in the specific cell types involved in the disease. Studies designed to address issues such as dose-dependence, time-course of the effects, and on the impact of the reciprocal stimulation of the two pathways will be needed.

Nevertheless, these efforts will not be sufficient if not paralleled by an active search for activators with the highest efficacy and better safety profiles. In the last years, different strategies have been employed to this goal: the repurposing of drugs already in use, searching in the world of nutraceuticals, the synthesis of new compounds, and in silico analyses. A combination of multiple approaches, such as those herein employed, might be a promising strategy leading to the development of new active molecules to counteract multifactorial diseases.

## 4. Materials and Methods

### 4.1. Cell Cultures

Purified cultures of OPs were obtained from Wistar rats (RRID: RGD_13508588; from Charles River, Lecco, Italy), as previously described [12], in accordance with the European Communities Council Directive N. 2010/63/EU and using the procedure approved by the Ministry of Health (authorization number: 152/2016-PR). Forebrains dissected from postnatal day 1 rat pups were mechanically dissociated. The cells obtained were seeded on poly-l-lysine-coated 60-mm-diameter culture dishes and grown for 10 days in Dulbecco’s modified Eagle’s medium (DMEM with high glucose) supplemented with 10% fetal bovine serum. OPs were obtained by mechanical detachment from the mixed cultures followed by 1-h incubation at 37 °C in culture flasks to minimize microglia contamination by removing adhering cells. OPs were seeded at a density of 6 × 10^4^ cells/cm^2^ on poly-l-lysine-coated 12-mm or 20-mm-diameter glass culture dishes or 96-well plates for immunocytochemistry, biochemistry, or video imaging. After 2 h, the culture medium was replaced with a chemically defined serum-free medium consisting of DMEM/Ham F12 (4:1) supplemented with 5.6-mg/mL glucose, 5-μg/mL insulin, 100-μg/mL human transferrin, 100-μg/mL bovine serum albumin, 0.06-ng/mL progesterone, 40-ng/mL sodium selenite, 16-μg/mL putrescine, 50-U/mL penicillin, 50-μg/mL streptomycin, 2-mmol/L glutamine, 10-ng/mL human recombinant Platelet-derived growth factor (PDGF-AA), and 10-ng/mL human recombinant basic fibroblast growth factor (bFGF) (PeproTech EC, Ltd., London, UK). Cell culture reagents were from Invitrogen (Milan, Italy) and Gibco (Thermo Fisher Scientific, Inc., Milan, Italy); other chemicals were from Sigma-Aldrich Italia (Milan, Italy).

### 4.2. Proliferation and Viability Assays

Cell viability was assessed by evaluating the ability of cells to reduce 3-(4,5-dimethyl thiazol-2-y1)-2,5-diphenyl tetrazolium bromide (MTT). The cells were incubated for 4 h in the presence of MTT, and then, dimethylsulfoxide (DMSO) was added to dissolve the dark blue crystals. A microplate reader (GDV, Rome, Italy) was used to measure the optical density by spectrophotometry, using a test wavelength of 570 nm and a reference wavelength of 630 nm. Optical densities were expressed as a percentage of the one measured in the control cultures, taken as 100%. The number of cells was determined by crystal violet (CV) proliferation assay. OP cells were incubated with the dye for 30 min at room temperature (RT), washed, solubilized in 1% SDS solution, and after 1 h at room temperature (RT), absorbance was read by a spectrophotometer at 595 nm. Values were expressed as a percent of the control cultures.

### 4.3. GSH Assay

Primary cultures of OP were treated in triplicate with DMF (3, 10, 30, and 100 μM). Replicate cultures were treated such that cells were exposed to a compound for 1, 6, or 24 h. The GSH levels were measured by employing a luciferase-based luminescence assay kit (GSH-GloTM Glutathione Assay, Promega, Madison, WI, USA, cat# V6911/2) following the manufacturer’s protocol.

### 4.4. Immunocytochemistry and Western Blot

For immunocytochemistry experiments, cells were fixed in 4% paraformaldehyde for 10 min at RT. Early OP differentiation was characterized by antigen expression using the monoclonal antibody O4 (IgM, hybridoma supernatants; diluted 1:5) and the fluorescein-conjugated goat anti-mouse IgM as the secondary antibody (1:200, Jackson ImmunoResearch Laboratories, Inc., West Grove, PA, USA). After fixation and permeabilization with 0.2% Triton X-100 for 10 min at RT, the cells were preincubated with 3% BSA in 0.1% Triton X-100/PBS solution for 1 h at RT. Then, the cells were incubated overnight at 4 °C with rabbit polyclonal anti-COX1 (1:100, Cat# ab45918, RRID:AB_944283, Abcam, Cambridge, UK), anti-PGC-1α (1:50, Cat# sc-13067, RRID:AB_2166218, Santa Cruz Biotechnology, Inc., Dallas, TX, USA), anti-MnSOD (1:600, Cat# SOD-111, RRID:AB_2315344, Stressgen Bioreagents Corp., Victoria, BC, Canada), anti-NRF2 (1:50, Cat# sc-13032, RRID:AB_2263168, Santa Cruz Biotechnology Inc., USA), or anti-HO1 (1:100, Cat# MA1-112, RRID:AB_2536823, Thermo Scientific Biotechnology Inc., New York, NY, USA) and mouse anti-mtTFA (more commonly known as TFAM; 1:500, Cat# sc-166965, RRID:AB_10610743, Santa Cruz Biotechnology Inc., USA) in the same pre-incubation solution. At the end of the primary antibodies incubation, cells were washed with PBS and incubated for 1 h at RT with goat anti-rabbit IgG-FITC (1:200; Jackson ImmunoResearch Laboratories, Inc., West Grove, PA, USA). Nuclei were stained using Hoechst 33258 (5 μg/mL for 20 min; Sigma Aldrich) and coverslips containing cells were mounted with Vectashield Mounting medium (Vector Laboratories, Burlingame, CA, USA). A Leica DM4000B fluorescence microscope equipped with a DFC420C digital camera and Leica Application Suite Software (260RI) were used for image acquisition (Leica, Wetzlar, Germany). Cells were counted in 10 microscopic fields of 0.18 mm^2^ per coverslip in duplicate for each condition from at least 3 independent experiments. ImageJ software (http://rsb.info.nih.gov/ij/) was used for immunofluorescence quantification, using the analysis of threshold fluorescence intensity within the regions of interest corresponding to single-cell profiles and for the colocalization of specific markers (Hoechst 33258 and NRF2) using the Pearson’s correlation coefficient [61].

For Western blot analysis, cells were homogenized on ice in RIPA buffer (phosphate-buffered saline, 1% NP-40, 0.5% sodium deoxycholate, 0.1% sodium dodecyl sulphate, and protease inhibitors), and centrifuged at 12,000× *g* for 20 min at 4 °C. An amount of 30 µg of protein was separated by 8–12% SDS-PAGE (Novex, Life Technologies, Monza, Italy). After transferring the proteins to polyvinylidene difluoride membranes, blots were incubated overnight at 4° C with the following antibodies: rabbit polyclonal anti-PGC1α (1:400, Cat# sc-13067, RRID:AB_2166218, Santa Cruz Biotechnology Inc., USA), anti-COX1 (1:100, Cat# ab45918, RRID:AB_944283, Abcam, UK), anti-MnSOD (1:600, Cat# SOD-111, RRID:AB_2315344, Stressgen Bioreagents Corp., Victoria, BC, Canada), anti-NRF2 (1:50, Cat# sc-13032, RRID:AB_2263168, Santa Cruz Biotechnology Inc., USA), or anti-HO1 (1:100, Cat# MA1-112, RRID:AB_2536823, Thermo Scientific Biotechnology Inc., USA), or mouse monoclonal anti-MBP (1:100, Cat#MAB382, RRID: AB_94971, Millipore, Milano, Italy) and mouse anti-α-actin (1:5000, Cat# A2228, RRID:AB_476697, Sigma Aldrich); the latter used as the internal control. After incubation with horseradish peroxidase (HRP)-conjugated antibodies polyclonal or monoclonal (1:4000; Jackson Immunoresearch, Milan, Italy), immunoreactive bands were revealed by an enhanced chemiluminescent substrate (ThermoFisher, Pierce, Scotland, UK). The bands, acquired with the ChemiDoc XRS imaging system (Bio-Rad, Milan, Italy), were analyzed using Image Lab 4.0 software (Bio-Rad).

### 4.5. Fluorescence Video Imaging

To measure the mitochondrial membrane potential (mMP), the potentiometric dye tetramethylrhodamine-ethyl ester perchlorate (TMRE) (Cat#87917 Lot#1350313, Sigma-Aldrich) was used at a final concentration of 30 nM (from 1-mM stock solution in DMSO). Cells were kept for 30 min in the presence of TMRE before recording to reach dye saturation. An excitation wavelength of 535 nm was applied, and the emission wavelength at 590 nm was recorded. To measure mMP, we chose mitochondria in cell extensions, because single mitochondria were only visible in this part of the cell. MitoSOX probe (Cat# M36008, Lot# 1721174, Thermo Fisher Scientific Inc.) was used to evaluate the mitochondrial superoxide. Cells were loaded for 30 min with the final concentration of 2-μM MitoSOX (from 5-mM solution in DMSO), and then, the solution was washed out right before fluorescence recording from each culture dish to avoid mitochondrial superoxide-independent MitoSOX oxidation. An excitation wavelength of 545 nm was applied, and the emission wavelength at 590 nm was recorded. An inverted microscope (Axiovert 135, Zeiss, Wetzlar, Germany) equipped with an oil immersion objective 40×, 1.35 NA (Olympus, Tokyo, Japan), was utilized for fluorescence video imaging. The appropriate excitation wavelengths were applied by means of a monochromator (Polychrome II; Till Photonics, Munich, Germany), and the emission light was collected by a CCD, cooled digital camera (PCO; Sensicam, Kelheim, Germany) and recorded on the hard disk of a PC. The Imaging Workbench 6.0 software package (Indec BioSystems, Santa Clara, CA, USA) was used for recording and analysis of the data.

The Mito Tracker Green probe (Cat#M7514 Lot#431620 Thermo Fisher Scientific Inc.) was used to measure the mitochondrial length. Cells were incubated with 40 -nM MitoTracker Green for 30 min. The fluorescence intensity, produced by the excitation wavelength 488 nm obtained by an argon ion laser and observed at emission wavelength 495–550 nm, was collected through a planapo objective (Olympus) 60× oil A.N.1.42 and recorded by a FV1000 confocal microscope (Olympus, Tokyo, Japan) equipped with a confocal spectral imaging system (Olympus Fluoview 1000). Images recorded had an optical thickness of 0.5 μm. Images were recorded at 640 × 640 pixels, 16 bits, corresponding to a dimension of 140.8 × 140.8 μm for each frame, and each pixel had the dimensions of 0.22 × 0.22 μm. Images were analyzed by NIH ImageJ software (http://rsb.info.nih.gov/ij/).

The solution used for loading and recording had the following composition (mmol/L): 140 NaCl, 5 KCl, 2.5 CaCl_2_, 1 MgCl_2_, 10 *D*-glucose, and 10 HEPES/NaOH (RT, pH 7.4, and 290 mOsm/L).

Data were analyzed offline by measuring the emission value within the regions of interest or along the line profiles. The averages of the amplitudes were calculated from a minimum of 3 experiments for each condition and are shown in bar graphs as mean ± SEM, with *n* = number of observations. All fluorescence signals were corrected for background before recording.

### 4.6. Reagents

Compounds 1 and 2 were synthetized according to previous procedures [24,39]. DMF (CAS number 624-49-7), pioglitazone (CAS number 112529-15-4), and curcumin (CAS number 458-37-7) were purchased by Sigma-Aldrich (Merck KGaA, Darmstadt, Germany). All compounds were solubilized in DMSO at stock concentrations of 10 mM, frozen (−20 °C) in aliquots, and diluted in culture medium immediately before use. 4-Hydroxy-TEMPO (4HT) (50 µM; CAS number 2226-96-2; Sigma-Aldrich, Merck KGaA, Darmstadt, Germany) and 2-µM zinc protoorphirin-9 (ZnPPIX) (Cas number 15442-64-5; Santa Cruz Biotechnology) were added 30 min before DMF.

### 4.7. Statistical Analysis

Data are shown in the graphs as mean ± SEM or as mean (horizontal line) and individual data points. Statistical significance was evaluated using Student’s *t*-test or 1-way analysis of variance (ANOVA). Numbers of independent experiments are indicated in the figure legends; *p* < 0.05 was accepted as significant. Analyses were performed using Stata™ 8.1 software (Stata Corporation, College Station, TX, USA) or Origin 7.5 (OriginLab, Northampton, MA, USA).

The experimental procedures and statistical analysis of the data presented in this study followed the methodologies and standards generally used in in-vitro studies. In all experiments, the sources of variability, as well as any residuals deriving from random errors of the replicates, were well-controlled, as demonstrated by the low SEM. Bonferroni correction was adopted to account for multiple comparisons. Six comparisons were considered (DMF vs. CTR, Pio vs. CTR, DMF vs. Pio, compound 1 vs. DMF, compound 1 vs. CTR, and compound 2 vs. CTR). The Shapiro Wilk test assessed the normality of the distribution.

## Supplementary Materials

The following are available online at https://www.mdpi.com/1422-0067/21/19/7216/s1, Figure S1. Effect of DMF on GSH levels.

## Abbreviations

*4HT*	4-hydroxy-tempo
*ARE*	Antioxidant responsive element
*ATP*	Adenosine triphosphate
bFGF	basic Fibroblast Growth Factor
CTR	Control
*COX1*	Complex IV core protein
*Curc*	Curcumin
*CuZn SOD*	Copper and zinc Superoxide Dismutases
CV	Crystal violet
*DMF*	Dimethyl-fumarate
DMSO	Dimethylsulfoxide
*GSH*	Glutathione
*HO-1*	Heme oxygenase 1
*IF*	Immunofluorescence
*Keap1*	Kelch-like ECH-associated protein 1
*MBP*	Myelin basic protein
*MFI*	Mean fluorescence intensity
*mMP*	Mitochondrial membrane potential
*MnSOD*	Manganese Superoxide Dismutase
*MTT*	3-(4,5-dimethyl thiazol-2-y1)-2,5-diphenyl tetrazolium bromide
*NFR2*	Nuclear factor-erythroid 2 (NF-E2)-related factor
*OL*	Oligodendrocyte
*OP*	Oligodendrocytes progenitor
PDGF-AA	Platelet-derived growth factor-AA
*PGC1a*	Peroxisome proliferator-activated receptor gamma coactivator 1-alpha
*Pio*	Pioglitazone
*PPAR-γ*	Peroxisome proliferator-activated receptor gamma
*PPRE*	PPAR-γ Response Element
*ROS*	Reactive Oxygen Species
*Rot*	Rotenone
*RT*	Room temperature
*RXR*	Retinoic x Receptor
*TFAM*	Mitochondrial transcription factor A
*TMRE*	Tetramethyl rhodamine ethyl ester
*TNF-a*	Tumor necrosis factor
*UCP2*	Uncoupling protein 2
*WB*	Western blot
*ZnPPIX*	HO-1 inhibitor Zn protoporphyrin IX
