# Supplementary figures and images for: NRF2 and PPAR-γ Pathways in Oligodendrocyte Progenitors: Focus on ROS Protection, Mitochondrial Biogenesis and Promotion of Cell Differentiation
Source: Int J Mol Sci. 2020 Sep 29;21(19):7216. doi: 10.3390/ijms21197216 (PMC7583077; doi:10.3390/ijms21197216)

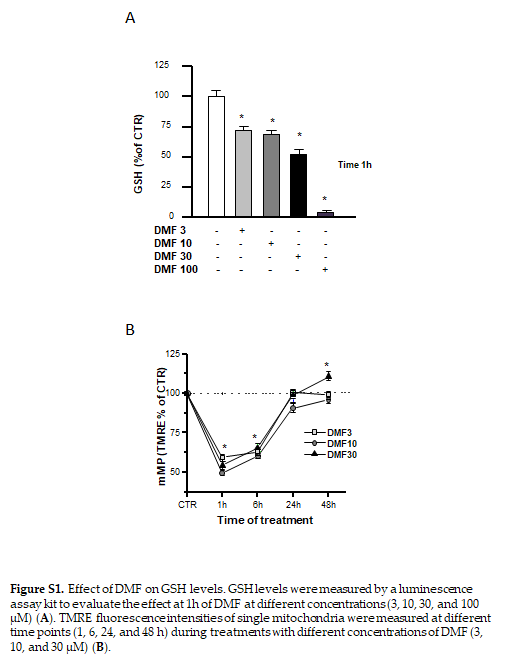

Supplement: Supplementary file 1 [file ijms-21-07216-s001.zip › ijms-912248-supplementary.tif]
